# Retinal detachment with multiple macrocysts in Stickler syndrome: case report and review of the literature

**DOI:** 10.3389/fmed.2024.1367281

**Published:** 2024-03-26

**Authors:** Guina Liu, Ming Hu, Chengcheng Cai, Xiaoshuang Jiang, Fang Lu

**Affiliations:** Department of Ophthalmology, West China Hospital, Sichuan University, Chengdu, China

**Keywords:** Stickler syndrome, rhegmatogenous retinal detachment, multiple macrocysts, COL2A1, case report

## Abstract

**Background:**

Stickler syndrome is a hereditary connective tissue disorder associated with ocular, orofacial, musculoskeletal, and auditory impairments. Its main clinical characteristics include retinal detachment, hearing loss, and midface underdevelopment. In clinical practice, macrocyst is rarely reported in retinal detachment cases with Stickler syndrome.

**Case presentation:**

We report the case of a 7-year-old child who developed a rhegmatogenous retinal detachment (RRD) in the right eye, accompanied by multiple peripheral macrocysts. The detachment was successfully surgically repaired with vitrectomy, retinal laser photocoagulation, cryotherapy and silicone oil tamponade. During the operation, a mini-retinectomy in the outer layer of each macrocyst was made for vesicular drainage and retinal reattachment. Genetic testing identified a pathogenic point mutation variant (c.1693C>T; p.Arg565Cys) in exon 26 of the COL2A1 gene. Six-months after the operation, the retina remained attached with improvement of best corrected visual acuity to 20/200.

**Conclusion:**

Patients with Stickler syndrome may develop RRD of different severity. Macrocyst is rarely reported in previous literature of Stickler syndrome. In this case report, we share our experience in treating with multiple macrocysts in RRD and emphasize the importance of periodic follow-up for patients with Stickler syndrome.

## Introduction

Stickler syndrome is a hereditary connective tissue disorder leading to joint problems, hearing difficulties, ocular abnormalities and midfacial hypoplasia (1). Typical ocular manifestations include progressive myopia, degeneration of the vitreous body, and secondary rhegmatogenous retinal detachment (RRD) (1, 2). Patients with Stickler syndrome, owing to collagen synthesis disorder, are at high risk to develop RRD (3). Moreover, RRD often results in poor outcomes during childhood (4, 5). Pediatric RRD, due to its rarity, may easily be neglected or diagnosed lately, ultimately resulting in irreversible vision loss (6, 7). Recognizing pediatric RRD is crucial, particularly among patients with Stickler syndrome.

We present an uncommon presentation of RRD observed in Stickler syndrome. A 7-year-old boy with severe visual loss in his right eye was referred to our ophthalmology clinic. The patient was diagnosed with Stickler syndrome type I with secondary RRD in his right eye, accompanied by unusual multiple inferior peripheral macrocysts. RRD is commonly reported as a severe ocular complication in Stickler syndrome, but the presence of multiple macrocysts is quite rare. In this article, we aim to present this particular case of RRD with multiple macrocysts and discuss the therapeutic interventions employed.

## Case presentation

**Figure 1:**
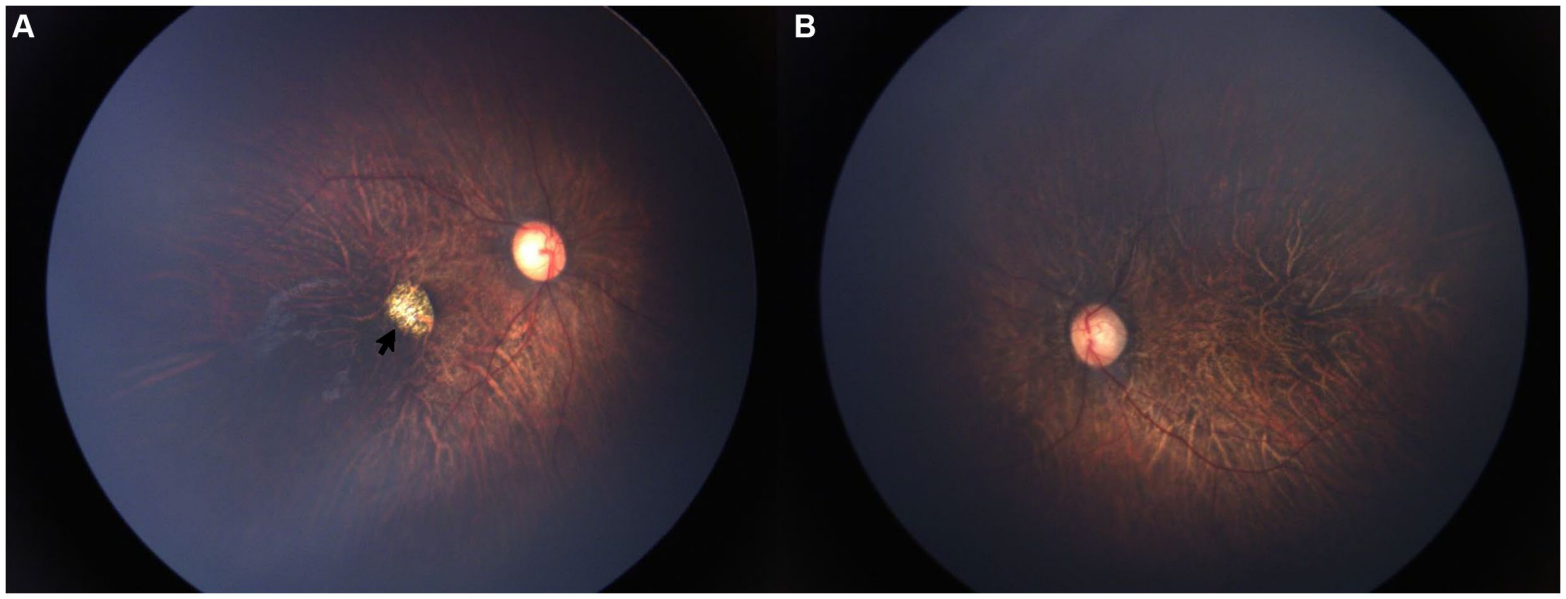
Fundus color images 2 years before. Color fundus images showed high myopia retinopathy in both eyes and macular coloboma (black arrow) in the right eye. **(A)** OD, **(B)** OS.

On 12 May 2021, a five-year-old boy initially came to our hospital for high myopia. The best corrected visual acuity (BCVA) was limited to FC/30 cm in right eye and 20/100 in left eye. Cup-to-Disc (C/D) vertical was 0.6 in the right eye and 0.5 in the left, and vitreous degeneration was found in both eyes. Fundus color photography showed tigroid fundus in both eyes and macular coloboma in the right eye (Figure 1). Moreover, midfacial hypoplasia was noted in the patient, with a normal hearing. No relevant family history was reported in this case. The boy was delivered by cesarean section at a gestational age of 36 weeks, weighing 2,800 g at birth. He required oxygen inhalation shortly after birth, though the specific details were unavailable. Throughout the entire pregnancy, there was no history of congenital infection. A diagnosis of Stickler Syndrome Type I was highly suspected and DNA sequencing test was recommended, but declined by his parents. Finally, periodic fundus examination was recommended. Unfortunately, due to the COVID-19 pandemic, periodic follow-up was not available.

**Figure 2:**
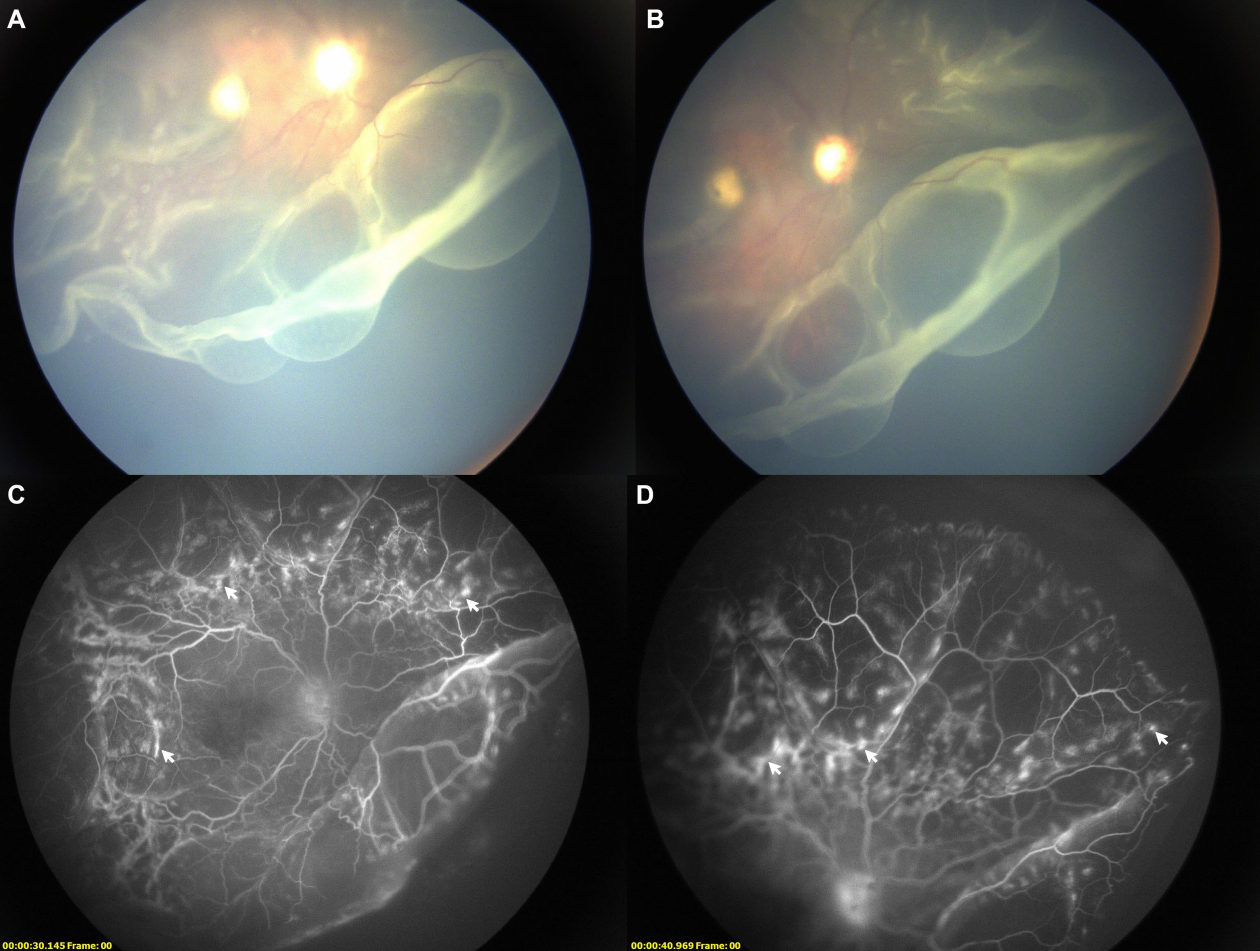
Images of right retinal detachment with multiple macrocysts. **(A,B)** Fundus color images showing retinal detachment with multiple macrocysts; **(C,D)** FFA images showing slight leakage of fluorescein in retinal vessels (white arrow).

Two years later, he was referred to our ophthalmology clinic for a severe decreasing of vision in his right eye. Following comprehensive fundus examinations including indirect ophthalmoscope, ultrasound B scan, color retinal photograph and fundus fluorescein angiography (RetCam 3, Clarity Medical Systems, Inc., CA, United States), a RRD with dialysis of ora serrata was observed in his right eye. The BCVA was limited to light perception in the right eye and 0.2 in the left eye. Intraocular pressure (IOP) was 20.1 mmHg in right eye and 21.2 mmHg in left eye. C/D vertical was 0.6 in the right eye and 0.5 in the left. Signs of high myopia were observed in both eyes and the macular coloboma was still present in the right eye. RRD with dialysis of ora serrata was noted in the right eye. Unlike other cases of RRD associated with Stickler syndrome, multiple retinal macrocysts were observed in the inferior retina and fluorescein leakage in the retinal vessels was observed in the right retina (Figure 2). DNA sequencing test identified a pathogenic point mutation variant (c.1693C>T; p.Arg565Cys) in exon 26 of the COL2A1 gene, confirming a diagnosis of Stickler syndrome type I. His parents underwent the same genetic test, but no variant was found indicating that it arose as a *de novo* mutation in the child.

After comprehensive evaluation, pars plana vitrectomy was performed after posterior vitreous detachment, and photocoagulation and cryotherapy were applied to the flattened retina to attach the neuroretina to the retinal pigment epithelium. Finally, silicon oil was injected into the vitreous. A mini-incision in the outer layer of each macrocyst was made for vesicular drainage and retinal reattachment. The procedure was performed by an experienced vitreoretinal surgeon (F.L.). The patient was instructed to maintain a face-down position after surgery for 1 month.

**Figure 3:**
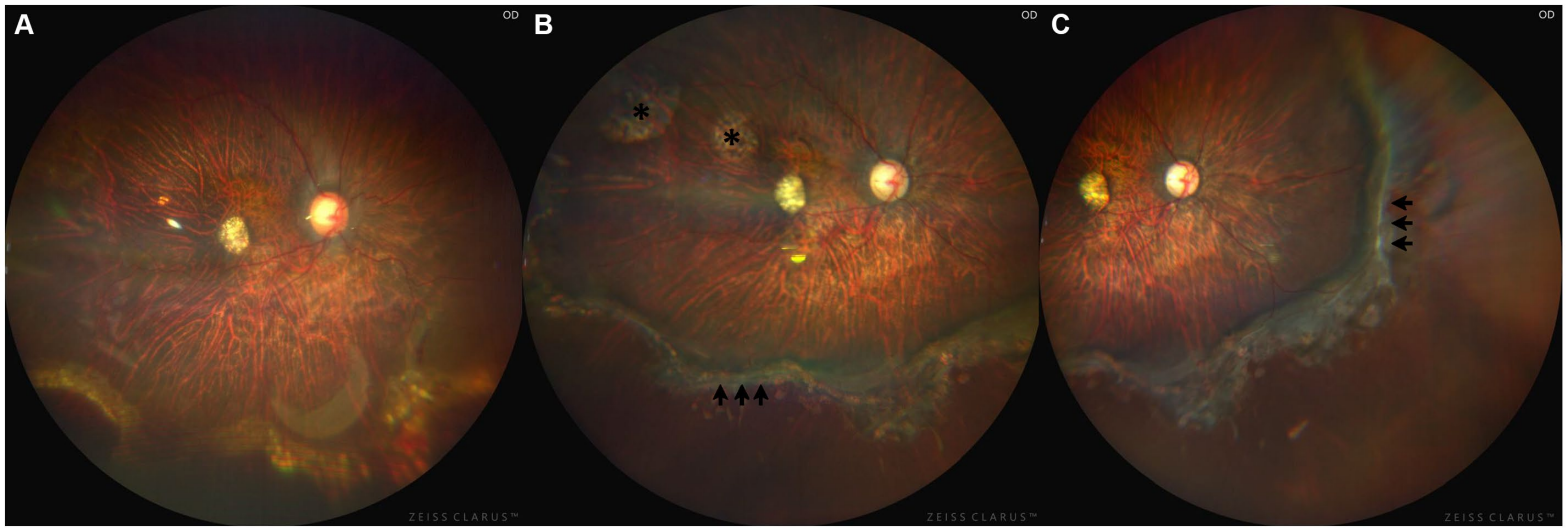
Fundus color images of right eye after operation. **(A)** Fundus color image showed the retina kept attached for 1 week after operation and the silicone oil was in the vitreous cavity. **(B)** Fundus color image showed laser spots (black arrows) for 4 months after operation and silicone oil was removed. The laser spots marked with the asterisk (*) are scars followed by laser treatment during vitrectomy. **(C)** Fundus color image showed the retina kept attached for half a year after operation.

One-week after the operation, fundus color images (CLARUS 500, Carl Zeiss, Dublin, United States) showed reattached retina with silicone oil tamponade in the vitreous cavity (Figure 3A). One-month after the operation, this right eye received additional laser coagulation on peripheral retina. Four-months after the operation, the silicone oil was removed (Figure 3B) and BCVA was 20/1,000 after surgery. Six-months after the operation, a fundus examination confirmed that his right retina remained attached and BCVA improved to 20/200 (Figure 3C). During the follow-up, IOP of the right eye maintained about 10~21 mmHg and the C/D ratio remained stable.

## Discussion and conclusions

Stickler syndrome is likely the most prevalent genetic cause of pediatric RRD, with approximately 60% of Stickler syndrome patients developing RRD at some point in their lifetime (3). Unlike other forms of RRD, those associated with Stickler syndrome are particularly complex and challenging to manage due to giant retinal tears and an abnormal vitreoretinal interface (8). Adding to the complexity, our patient exhibited giant retinal tears with multiple macrocysts. In most cases, retinal detachment is reported to be associated with only a single retinal macrocyst (9–11) and macrocyst formation predominantly occurred in adults (12, 13). A clinical presentation featuring multiple macrocysts has only been reported once before, in an 11-year-old boy with Stickler syndrome, but the variant gene was different (COL11A1 mutation) (14).

Retinal macrocysts are rare entities reported in 1%–3% of retinal detachments (15). The underlying cause of multiple macrocysts remains unclear. Liu et al. (13) clarified that these macrocysts were not true cysts but rather formations resulting from the splitting of retinal layers and often did not cause ocular symptoms or affect visual acuity (15). In this case, the presence of multiple retinal macrocysts may have been due to long-standing chronic RRD that lacked timely diagnosis and treatment, similar with the case reported by Venkatkrish et al. (14). Given the limited number of patients, a deeper understanding of multiple retinal macrocysts in RRD is needed in future studies.

For the majority of retinal macrocysts, specific intervention was not necessary during the repair of retinal detachment (12). Surgical drainage was required if the cyst obstructed the closure of the primary retinal break (12, 16). In this case, a mini-retinectomy was made in the outer layer of each retinal microcyst to facilitate the drainage of fluid in macrocysts and minimize the opening of cysts. This strategy was selected due to the inferior peripheral location of the retinal macrocysts, poor cooperation of face down position due to the young age of the patient. Furthermore, opting for the outer layer of the retina instead of cutting on the inner layer reduced the need of extensive laser photocoagulation on these macrocysts.

Patients with Stickler syndrome are at a high risk for retinal detachment, facing a lifetime risk of approximately 65% (17, 18). Considering the early onset of RRD in Stickler syndrome and its challenging visual prognosis, some retinal specialists recommend prophylactic interventions like 360-degree cryotherapy (18), argon laser photocoagulation (19), or scleral buckling (20) to prevent potential retinal tears and RRD. However, the available data on the efficacy of these prophylactic procedures lacks robust comparative analysis to identify the optimal approach for Stickler syndrome, and there are no consensus or guidelines on prophylactic treatment (21). As a result, no preventive treatment was administered to the left eye in this case. Instead, regular fundus examinations and avoidance of vigorous exercise were suggested. Fortunately, his left eye kept stable during the follow-up period.

Different from other cases of RRD in Stickler syndrome (14, 22), our patient presented with macular coloboma in right eye. Congenital macular coloboma is a relatively rare condition involving 0.5–0.7/10,000 births (23). It is most seen as isolated cases without inheritance pattern though rarely autosomal dominant and other inheritance patterns have been reported (24). In this case, we excluded the infectious factors. We hypothesized it might be associated with the p.Arg565Cys mutation in COL2A1 for a previous study that reported individuals with Stickler syndrome carrying this mutation exhibited more severe foveal hypoplasia, macular degeneration, and extensive retinal degeneration across two families (25).

There were limitations in this case report. First of all, the absence of a larger number of similar cases limits our understanding of the prevalence and cause of multiple retinal macrocysts in pediatric chronic RRD. Secondly, current examination outcomes and prior literature do not provide a clear explanation for the occurrence of macular coloboma. Additionally, therapies were selected based on the patient’s condition and the ophthalmologist’s experience, in the absence of guidelines or consensus due to the rarity of the presentation. Finally, prolonged follow-up is crucial for a comprehensive prognosis of retinal structure and functionality.

In conclusion, we reported a case of a 7-year-old child with Stickler syndrome who developed RRD with multiple macrocysts. Conventional RRD repair and vesicular drainage from the outer layer were performed. Six months post-operation, the retina kept attached and the BCVA improved to 20/200. Despite the high risk of RRD for the other eye, no preventive treatment was taken because of the uncertain efficacy of prophylactic treatment. In this case, the cause of the multiple retinal macrocysts remains unclear and needs further investigations to understand their pathogenesis. For patients with Stickler syndrome, it is crucial to avoid vigorous exercise, undergo periodic fundus examinations and ask for ophthalmic help immediately if severe visual loss occurs.

## Data availability statement

The original contributions presented in the study are included in the article/supplementary material, further inquiries can be directed to the corresponding author.

## Ethics statement

The studies involving humans were approved by Ethics Committee on Biomedical Research, West China Hospital of Sichuan University. The studies were conducted in accordance with the local legislation and institutional requirements. Written informed consent for participation in this study was provided by the participants’ legal guardians/next of kin. Written informed consent was obtained from the individual(s), and minor(s)' legal guardian/next of kin, for the publication of any potentially identifiable images or data included in this article.

## Author contributions

GL: Formal analysis, Writing – original draft, Writing – review & editing. MH: Writing – review & editing. CC: Formal analysis, Investigation, Writing – original draft. XJ: Writing – review & editing. FL: Funding acquisition, Writing – review & editing.
